# Persian version of the brief Older People’s Quality of Life questionnaire (OPQOL-brief): the evaluation of the psychometric properties

**DOI:** 10.1186/s12955-020-01586-8

**Published:** 2020-10-07

**Authors:** Awat Feizi, Zahra Heidari

**Affiliations:** 1 Department of Biostatistics and Epidemiology, School of Health, Isfahan University of Medical Sciences, Isfahan, Iran; 2 Cardiac Rehabilitation Research Center, Cardiovascular Research Institute, Isfahan University of Medical Sciences, Isfahan, Iran; 3 Psychosomatic Research Center, Isfahan University of Medical Sciences, Isfahan, Iran

**Keywords:** Quality of life, Older adults, Validity, Reliability, Psychometrics

## Abstract

**Background and objective:**

Quality of life (QoL) is a multi-dimensional concept and its assessment is one of important themes of care for older people. Assessing QoL in older people needs specific scales. The aim of this study was to culturally adapt and investigate the psychometric properties of the Persian version of brief Older People’s Quality of Life questionnaire (OPQOL-brief) in an Iranian older population.

**Methods:**

This methodological cross-sectional study was conducted among 525 Persian-speaking older people (aged 60 and over), living in Isfahan, Iran. Translation of the OPQOL-brief questionnaire was performed using forward–backward method. Test–retest reliability was evaluated through Intra Class Correlation (ICC) coefficient and internal consistency by using Cronbach’s α. Construct validity was investigated by using exploratory factor analysis (EFA), confirmatory factor analysis (CFA), and Latent class analysis (LCA). Criterion, convergent and discriminant validities were also assessed.

**Results:**

Persian version of the OPQOL-brief showed good test–retest reliability (ICC = 0.842, 95% CI = 0.73–0.91; *P* < 0.001). Persian OPQOL-brief scale demonstrated high internal consistency (Cronbach’s α = 0.83). It showed good discriminant validity and differentiated old patients from healthy older individuals (*P* < 0.001). Construct validity based on EFA led to extraction of three dimensions (“socioeconomic”, “emotional”, and “physical” well-being) and the CFA confirmed the adequacy of extracted construct from EFA (CFI = 0.909, PCFI = 0.52, PNFI = 0.5, CMIN/DF = 3.012, and RMSEA = 0.08). LCA classified participants into three classes in terms of QoL level (low (16%), middle (67%), and high (17%)). Criterion validity and convergent validity revealed significant positive correlations between OPQOL-brief and physical and psychological dimensions of the SF-36.

**Conclusion:**

The Persian version of the OPQOL-brief is a reliable and valid instrument for assessing QoL with applicability in a broad range of older Persian language population.

## Introduction

The world's ageing population is growing dramatically as a result of longer life expectancy, improved living conditions and reduced fertility rates [1, 2]. According to World Population Prospects, adult population aged 60 and over in the world was 962 million in 2017 and it is expected to increase to 2.1 billion in 2050 [3]. According to the statistical data from population and housing census in 2011, Iran had 8.2% of people aged ≥ 60 years, and predicted that this rate will increase to 25.1% in 2061 [1, 4, 5]. The increase in the ageing population and corresponding rises in chronic illness is one of the most important public health challenges in the high-income countries [1, 6, 7]. In particular, it is associated with higher demand for, expenditure on, health and aged care services [8, 9]. Therefore, it is argued that if ageing is a challenge for high-income countries, then it is a significant challenge for countries with low and middle-income, such as Iran [6, 7, 10].

Various illnesses may be seen in older adults due to reduce physical and cognitive function in ageing, which all affect inversely on the quality of life (QoL) [3, 11]. QoL is a multidimensional concept and based on the World Health Organization definition is related to culture, value system in which they live, their goals, expectations, standards, concerns, and personal interests [5, 12]. It has been shown that QoL is a strong predictor of adverse health outcome, such as death in older people, and its evaluation is an important point for policymakers [3, 7, 13]. QoL in old age covers a variety of dimensions including: health condition and functional ability, sense of being useful for other people, social relationships, social support, financial situation, and the quality of housing [14, 15]. To properly evaluate older people’s quality of life, it is necessary to consider all various physical, mental, and social aspects of QoL using an appropriate scale [1, 16].

There are several instruments for measuring QoL in older people [1, 17–21]. Among the developed tools for evaluating QoL in older adults, OPQOL-35 is one the most popular instrument. It has 7 dimensions including: "life overall", "health", "social relationships/leisure and social activities", "independence, control over life, freedom", "home and neighborhood", "psychological and emotional well-being", "financial circumstances", and "religion/culture" [21]. Although OPQOL-35 provides comprehensive information about quality of life of older people in both healthy population and old patients [6]; however, OPQOL-35 is a long questionnaire to perform in the research projects and clinical practice [3]. There is a growing interest in research to create balance between scale length and levels of psychometric acceptability. It is believed that working with short scales can result in a high level of measurement accuracy [7]. Hereby, in 2013, Bowling et al. developed OPQOL-brief which contains all domains of OPQOL-35 except religion/culture [3]. The OPQOL-brief has only 13 items and showed acceptable levels of reliability and validity in older English people. It needs less time to complete and reduces research burden [3].

Since the QoL of older people is becoming an important public health issue, measuring QoL of the older adults can be helpful for planning health program in future [6]. To the best of our knowledge, there are only two Persian validated questionnaires for assessing QoL among older people in Iran i.e. CASP- 19 and OPQOL-35 scales [1, 6]. Given the value of the OPQOL-brief for the evaluation of the quality of life in older adults, this study aimed to determine the validity and reliability of the Persian version of OPQOL-brief among Persian ageing people. This instrument is short, comprehensive, quick and practical.

## Methods and materials

### Study design and participants

This methodological cross-sectional study was conducted among 525 Persian-speaking adults aged 60 years old and over living in Isfahan, a large city in central Iran, in 2019. The participants were selected from urban health centers of Isfahan through multistage cluster sampling. First, we selected randomly 5 and 7 urban health centers as the second-stage clusters from Isfahan health centers I and II (two main clusters), respectively. Then in each selected health center, older adults who fulfilled our inclusion criteria were identified. The inclusion criteria to the current study were aged 60 years and over, oral feeding (ability to eat and drink like a normal person), permanent resident of Isfahan city. The exclusion criteria were as follows: hospitalization during the last 3 months, history of major surgery, amputation, affecting with major cognitive problems and physical illness at the time of interview and stay in nursing homes. Therefore, participants mental and physical conditions were not such a way that they needed to have a nurse at home for dealing with their clinical conditions. We explained the purposes of study to the all eligible participants, and then invited them to participate in our study. Finally, 525 older adults agreed to participate in the study. Those people who agreed to participate in our study were invited to attend in a structured interview by trained interviewers in the health centers. All participants received enough information about the study and oral informed consent was obtained from them. The study protocol was approved by the Ethics Committee of Isfahan University of Medical Sciences (IR.MUI.RESEARCH.REC.1397.068; Project Number: 197066).

### Procedures

#### Older people’s quality of life-brief questionnaire (OPQOL- brief)

Bowling et al. [7] developed a questionnaire to measure QoL of older people. It was originated from OPQOL-35 questionnaire which has been validated in older adults in Britain [7]. This scale asked participants their level of agreement with 13 statements such as, "I am healthy enough to get out and about", or "I feel safe where I live”. Each statement has five-point Likert scale ranged from 1 (‘strongly disagree’), 2 (‘disagree’), 3 (‘neither agree nor disagree’), 4 (‘agree’), and 5 (‘strongly agree’). The total score of OPQOL-brief ranges from 13 to 65; and higher scores indicates higher QoL. It is a validated instrument with acceptable reliability (Cronbach’s α = 0.856) [7].

#### Translation

Methodology recommended by Beaton et al. was followed to translate the OPQOL-brief from English into Persian language [22]. Two independent professional translators translated the items into Persian (forward translation). One of them was aware of the concept of the items being translated, and the second translator was unaware of the items being examined in the original English instrument. Then a consolidated forward version was adopted by the current study's researchers (Z.H. and A.F.) and both translators. This questionnaire then was backward translated into English by two bilingual translators to compare with the original one with respect to conceptual equivalence. After a careful review by researchers (Z.H. and A.F.) necessary changes were made and the provisional Persian version of the OPQOL-brief questionnaire was provided. In general, there were no difficulties in translated questionnaire. Consequently we performed content validity by calculating Content Validity Index (CVI) and Content Validity Ratio (CVR). The CVI measures simplicity, relevance and clarity of each item in relation to the construct evaluated by the scale. We requested the eight professionals (4 doctoral Gerontology, and 4 epidemiologist and biostatistician with the experience of working by older people) to evaluate the simplicity, relevance and clarity of the Persian OPQOL-brief items on a 4-point rating scale. For example, the experts assessed the relevance of the items using: (1) not relevant; (2) slightly relevant; (3) relevant; and (4) completely relevant. A CVI of ≥ 0.79 was considered acceptable for each item. The CVR assesses the necessity of each item. For calculating CVR, eight experts were asked to rate the essentiality of the Persian OPQOL-brief items on a three- point scale i.e. 1: unnecessary; 2: useful but unnecessary; and 3: necessary. A CVR of ≥ 0.75 was considered satisfactory for each item [23, 24]. Qualitative face validity involved the expert panel and older people who evaluated the OPQOL-brief questionnaire for difficulty, relevance, and ambiguity. Then the final Persian version of the OPQOL-brief was developed and used for evaluating the psychometric properties.

#### Psychometric analysis of the OPQOL-brief

In this study, psychometric properties of the OPQOL-brief including reliability (test–retest reliability and internal consistency), validity (construct validity, discriminant validity, criterion and convergent validity) were evaluated.

### Validity

#### Construct validity

The factor structure of the OPQOL-brief was explored using the EFA and CFA. We performed a cross-validation, splitting the sample into two subsamples randomly. EFA was performed on the first half sample (training sample; n = 257) based on the principal component extraction approach and the orthogonal Varimax rotation. Factors were retained for further analysis based on their natural interpretation and eigenvalues on the Scree plot. In this study, we retained factors with eigenvalues > 1 as cutoff and factor-item loadings values greater than 0.40, which could result in more interpretable factors and explain sufficient amounts of overall variation. The data viability for factorability was guided through Kaiser–Meyer–Olkin (KMO) measure of sample adequacy (Values > 0.7) and Bartlett’s test of Sphericity (*P* < 0.05) [25]. The final extracted factors were labeled based on the loaded items in each factor. The factor score for each sub-scale (factors) was computed by summing up items multiplied by related loading and assigned to each participant. Subsequently, we performed a CFA on the other subsample (validation sample; n = 268) to confirm the derived factor structure from EFA. Comparative Fit Index (CFI) ≥ 0.9, Parsimony Comparative Fit Index (PCFI) > 0.5, Parsimony Normed Fit Index (PNFI) > 0.5, Chi-square/ degree of freedom ratio < 3, and Root Mean Square Error of Approximation (RMSEA) < 0.08 were used to confirm goodness of fit of the CFA [26, 27].

The latent structure of the OPQOL-brief was also investigated by using LCA. This model examines the pattern of relations among a set of observed categorical variables (here items of the OPQOL-brief) and classifies similar individuals in terms of QoL level into homogeneous latent classes [28]. This leads participants within each latent class are highly similar to each other and uniquely different from the other classes across the set of evaluated items. Accordingly, comparisons can be made across latent classes with regard to QoL level. We fitted various LCA models with different number of latent classes. The best model (i.e. the optimal number of classes) was guided through model fit indices including Akaike's information criterion (AIC), the Bayesian information criteria (BIC), the sample size-adjusted BIC and entropy. For all information criteria except entropy, the lowest value indicates ‘best’ model, i.e. the optimal number of classes in the current study [28].

#### Discriminant validity

Discriminant validity was assessed based on the OPQOL-brief ability to discriminate between healthy older individuals and older adults suffering from mental and physical illnesses in terms of QoL level. We hypothesized that the quality of life of a person with mental/physical illnesses was significantly different from that of a person without it. This hypothesis also applies to the dimensions of quality of life. We compared the total score of quality of life as well as its three dimensions (Socioeconomic well-being, Emotional well-being and Physical well-being) between groups with the above characteristics. The validity of the measure is supported if mean of the QoL levels is significantly different between two groups. We tested difference between two groups (older individuals with and without the illnesses) using independent Student’s t‑test.

#### Criterion validity and convergent validity

Criterion validity was assessed using Pearson correlation coefficients between the score of each OPQOL sub-scale and the physical and psychological dimensions of the 36-Item Short Form Health Survey (SF-36). The SF-36 is a general quality of life instrument that measures eight components: physical functioning (PF), role limitations due to physical health (RP), body pain (BP), general health (GH), mental health (MH), role limitations due to emotional problems (RE), vitality (energy/fatigue) (VT), and social functioning (SF). The first 4 components were also used to calculate physical component of QOL and the last 4 components to emotional component of QOL. The validity and reliability of the SF-36 has been evaluated in the Iranian population previously [29].

Convergent validity was assessed using Pearson correlation coefficients between the score of each OPQOL sub-scale and total score of Pitsburgh Sleep Quality Index (PSQI) and Insomnia Severity Index (ISI) questionnaires. PSQI was used to assess self-reported sleep quality over 1 month [30]. The PSQI consisted of 7 components including subjective sleep quality, latency, sleep duration, efficiency, sleep disturbance, use of sleep medications, and daytime dysfunction. The validity and reliability of PSQI were evaluated by Farrahi Moghaddam et al. (Cronbach’s α = 0.77) in Iran [31]. ISI was used to measure the participant’s perception of his/her insomnia. The total score of ISI ranges from 0 to 28. It is a validated instrument with acceptable internal consistency (Cronbach’s α = 0.83) [32].

### Reliability

To investigate internal consistency and test–retest reliability, we recruited 50 older individuals aged 60 years old and over. The participants were requested to participate in two interviews at two separate days with a 10 days interval. The first interview was conducted as face to face in health centers and the second interview was a telephone interview. All interviews performed by trained interviewers. To evaluate test–retest reliability, the ICC coefficient using two-way mixed model, along with 95% confidence was computed. The coefficient more than 0.70 was considered as excellent stability [33]. Internal consistency was evaluated using Cronbach’s α coefficient. The values between 0.70 and 0.95 were conventionally considered as satisfactory internal consistency [33]. Data collected on the pilot sample in the first administration of the OPQOL-brief measure was used to evaluate internal consistency. Ceiling and floor effects were assessed on the first administration of the OPQOL-brief to determine content validity.

#### Other measurements and statistical analysis

A comprehensive questionnaire was used to collect information about sociodemographic (e.g., age, sex, marital status, level of education, job, income level), history of smoking, and health-related characteristics such as history of physical and mental illnesses (e.g., hypertension, cardiovascular, diabetic, osteoporosis, arthritis, cancer, depression). In this paper, quantitative and qualitative variables were expressed as mean (standard deviation (SD)) and number (precent), respectively. Data analyses were performed using SPSS (version 16; SPSS Inc., Chicago, IL, USA) and R free statistical software version 3.2.2.

## Results

### Content and face validity

The expert panel checked the difficulty, relevance, and ambiguity of the wording and phrasing of the Persian OPQOL-brief items. The CVI ranged between 0.80 and 1.00 for all items of the Persian OPQOL-brief. In addition, the CVR ranged between 0.75 and 1.00 for all items. Consequently, no items were deleted (Table 1).

**Table 1.** Relevance, simplicity, clarity, Item Content Validity Index (I-CVI), and Content Validity Ratio (CVR) values of the Persian OPQOL-brief questionnaire

Items	Relevance	Simplicity	Clarity	I-CVI	CVR
1. I enjoy my life overall	1	1	0.875	0.958	1
2. I look forward to things	1	0.875	0.875	0.917	0.75
3. I am healthy enough to get out and about	1	1	1	1	0.75
4. My family, friends or neighbours would help me if needed	1	1	1	1	1
5. I am healthy enough to have my independence	1	1	1	1	0.75
6. I can please myself what I do	1	1	0.875	0.958	0.75
7. I feel safe where I live	1	1	1	1	1
8. I get pleasure from my home	1	1	0.875	0.958	0.75
9. I take life as it comes and make the best of things	1	1	1	1	1
10. I feel lucky compared to most people	1	1	1	1	1
11. I have enough money to pay for household bills	1	1	1	1	1
12. I have social or leisure activities/hobbies that I enjoy doing	1	0.875	0.875	0.917	1
13. I try to stay involved with things	1	1	1	1	0.75

### Participant characteristics

A total of 525 adults aged 60 years old and over participated in this study. Table 2 shows the distribution of the personal, sociodemographic, and health-related characteristics of the participants. Mean age ± SD of the participants was 69.15 ± 6.38 years and 51% were female gender. Twenty two point two percent (22.2%) of participants were actually illiterate, and 7.1% of them had academic level of education. Only 4.8% of participants had adequate income. Approximately half (47.9%) of the participants were living with his/her spouse, and 43.5% were sharing the household with his/her spouse and unmarried children. Only, 6.7% of older individuals were currently employed, and 5.6% of them were current smoker. A bit more than half (54.2%) of the participants reported that they suffered from hypertension, and 30% suffered from cardiovascular disease. In addition, depression, anxiety and sleep disorders were reported by 24%, 38.5% and 30.6% of participants, respectively (Table 2).

**Table 2.** General characteristics of the studied population

Characteristics	Mean (SD) or Number (percent) (n = 525)
Age (years)	69.15 (6.38)
*Gender*	
Female	268 (51.0)
Male	257 (49.0)
*Educational level*	
Illiterate	115 (22.2)
Ability of reading and writing	68 (13.1)
Primary school	153 (29.5)
Under diploma	80 (15.4)
Diploma	65 (12.5)
Academic	37 (7.1)
*Current job*	
Employed	35 (6.7)
Retired	224 (42.8)
Housekeeper	230 (44.0)
Unemployed	27 (5.2)
Other	7 (1.3)
*Marital status*	
Single	3 (0.6)
Married	484 (93.6)
Widow	30 (5.8)
*Income level*	
Inadequate	233 (45.4)
Middle	256 (49.9)
Adequate	24 (4.7)
*Type of house*	
Rental	28 (5.8)
Owner	452 (94.2)
*Housemate*	
Spouse	250 (47.9)
Spouse and unmarried children	227 (43.5)
Spouse and married children	34 (6.5)
Single	11 (2.1)
*Supporting in daily activities by family*	
Yes	93 (18.0)
No	33 (6.4)
No need	391 (75.6)
*Smoking*	
Nonsmoker	436 (84.3)
Former smoker	52 (10.1)
Current smoker	29 (5.6)
Number of children	5 (4–6)
*Physical illnesses*	
Hypertension (yes)	283 (54.2)
Cardiovascular (yes)	157 (30.0)
Osteoporosis (yes)	221 (43.3)
Diabetic (yes)	188 (36.2)
Arthritis (yes)	269 (51.5)
Digestive diseases (yes)	151 (29.0)
*Mental illnesses*	
Depression (yes)	126 (24.0)
Anxiety (yes)	202 (38.5)
Alzheimer and cognitive impairments (yes)	103 (19.7)
Parkinson (yes)	43 (8.2)
Sleep disorders (yes)	157 (30.6)

### Construct validity

**Fig. 1:**
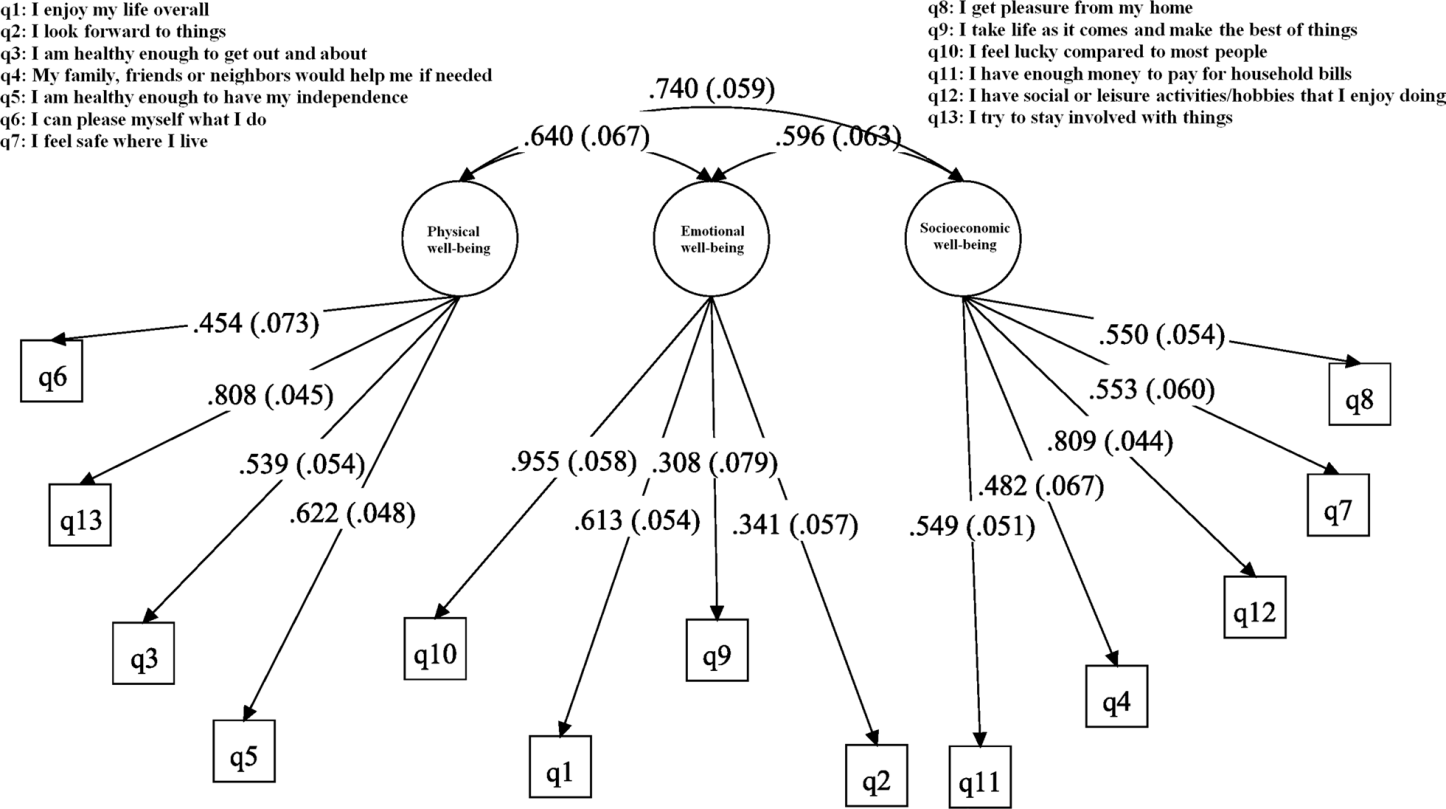
Confirmatory factor analysis testing the extracted construct from EFA on the Persian OPQOL-brief items

Construct validity was evaluated by using EFA, CFA and LCA. During EFA a KMO value of 0.772 and *P* < 0.05 for the Bartlett’s test confirmed the data viability for factorability. EFA with Varimax rotation extracted three factors from the OPQOL-brief measure which were labeled as “socioeconomic well-being”, “emotional well-being”, and “physical well-being” accounting for 20.9%, 19.8% and 17.2% of total variance, respectively. Table 3 provides the factor loadings of 13 items of the OPQOL-brief measure for three extracted factors from the EFA. The results obtained from the CFA indicated a good fit for data as follows: CFI = 0.909, PCFI = 0.52, PNFI = 0.5, CMIN/DF = 3.012, and RMSEA = 0.08 were confirmed goodness of fit of factor model also all items loaded significantly on their respective factors (Fig. 1).

**Table 3.** Factor loadings of the Persian OPQOL-brief to assess construct validity

Items	Extracted factors^a^		
	Socioeconomic well-being	Emotional well-being	Physical well-being
I get pleasure from my home	0.762		
I feel safe where I live	0.750		
I have social or leisure activities/hobbies that I enjoy doing	0.662		
My family, friends or neighbors would help me if needed	0.595		
I have enough money to pay for household bills	0.538		
I look forward to things		0.826	
I take life as it comes and make the best of things		0.757	
I enjoy my life overall		0.659	
I feel lucky compared to most people		0.577	
I am healthy enough to have my independence			0.889
I am healthy enough to get out and about			0.860
I try to stay involved with things			0.509
I can please myself what I do		0.542	0.412
Variance explained (%)*	20.9	19.1	17.2

^a^Exploratory factor analysis with Varimax rotation; Factor loadings < 0.4 are not shown for simplicity

*Variance explained resulted from factor analysis

Results of the LCA for classifying participants based on the 13 items showed that model with three classes has highest quality of fit to the data (BIC = 4236.7, AIC = 6022.01, SABIC = 5579.34 and entropy = 0.84). Additional file 1: Table S1 shows the percentage of the answers to the items of questionnaire in constructed classes. The nature of each class can easily be interpreted in terms of items' frequencies in each class. Accordingly, class 1 contains 16% of the study population with low level of quality of life and class 3, including 17% of participants, consisted of individuals with high level of quality of life. The second class included older individuals with middle level of quality of life (67%).

### Discriminant validity

Table 4 provides the results of discriminant validity based on comparing the total and sub-scales score of OPQOL-brief measure between older individuals with and without physical and mental illnesses. For example, mean OPQOL-brief score was 48.8 ± 6.92 in older individuals with depression and 53.7 ± 5.73 in individuals without it (*P* < 0.001). Similar results were obtained for three subscales, especially for socioeconomic well-being and physical well-being.

**Table 4.** Comparison of the total and sub-scales score of OPQOL-brief measure between older adults with and without physical and mental illnesses to assess the discriminant validity

Quality of life and its dimensions												
Physical and mental illnesses	Total score		*P* value	Socioeconomic well-being		*P* value	Emotional well-being		*P* value	Physical well-being		*P* value
Hypertension (n/y)	53.41 (6.82)	51.81 (5.93)	.004	21.35 (3.18)	20.71 (3.20)	0.024	19.81 (3.06)	19.39 (2.84)	0.104	16.40 (2.72)	15.73 (2.49)	0.003
Cardiovascular (n/y)	53.23 (6.03)	50.98 (6.90)	< 0.001	21.31 (3.03)	20.22 (3.47)	< 0.001	19.65 (2.96)	19.45 (2.88)	0.457	16.36 (2.35)	15.35 (2.98)	< 0.001
Osteoporosis (n/y)	53.50 (6.10)	51.30 (6.65)	< 0.001	21.17 (3.07)	20.74 (3.41)	0.135	20.06 (2.88)	18.96 (2.93)	< 0.001	16.42 (2.30)	15.59 (2.88)	0.001
Diabetic (n/y)	53.81 (5.69)	50.35 (6.99)	< 0.001	21.55 (2.76)	19.98 (3.68)	< 0.001	19.81 (2.93)	19.19 (2.94)	0.021	16.56 (2.29)	15.17 (2.87)	< 0.001
Arthritis (n/y)	53.48 (6.34)	51.69 (6.33)	0.001	20.99 (3.27)	20.98 (3.15)	0.969	20.20 (2.69)	19.01 (3.06)	< 0.001	16.46 (2.33)	15.68 (2.78)	0.001
Digestive diseases (n/y)	53.35 (5.97)	50.66 (6.37)	< 0.001	21.31 (2.97)	20.28 (3.31)	0.001	19.78 (2.96)	19.07 (2.81)	0.012	16.37 (2.34)	15.27 (2.85)	< 0.001
*Physical illnesses* (n/y)^a^	55.87 (5.78)	52.16 (6.34)	< 0.001	21.85 (2.75)	20.89 (3.23)	0.037	21.11 (2.77)	19.41 (2.91)	< 0.001	17.27 (2.04)	15.90 (2.63)	< 0.001
Depression (n/y)	53.73 (5.73)	48.80 (6.92)	< 0.001	21.61 (2.70)	19.04 (3.83)	< 0.001	19.85 (2.96)	18.73 (2.70)	< 0.001	16.39 (2.40)	14.95 (2.93)	< 0.001
Anxiety (n/y)	54.23 (5.41)	49.86 (6.89)	< 0.001	22.02 (2.41)	19.36 (3.60)	< 0.001	19.82 (2.97)	19.21 (2.86)	0.02	16.53 (2.32)	15.26 (2.85)	< 0.001
Alzheimer and cognitive impairments (n/y)	53.32 (5.78)	49.68 (7.01)	< 0.001	21.55 (2.81)	18.87 (3.33)	< 0.001	19.53 (2.99)	19.86 (2.66)	0.303	16.33 (2.36)	15.00 (3.01)	< 0.001
Parkinson (n/y)	52.95 (6.03)	47.95 (8.25)	< 0.001	21.20 (2.96)	18.62 (4.55)	0.001	19.64 (2.92)	18.95 (3.05)	0.140	16.21 (2.48)	14.16 (3.25)	< 0.001
Sleep disorders (n/y)	54.25 (5.21)	48.70 (7.02)	< 0.001	21.64 (2.61)	19.56 (3.89)	< 0.001	20.10 (2.77)	18.41 (2.84)	< 0.001	16.66 (2.15)	14.61 (3.01)	< 0.001
*Mental illnesses* (n/y)^b^	55.10 (5.11)	50.39 (6.56)	< 0.001	22.30 (2.33)	19.89 (3.41)	< 0.001	20.07 (2.95)	19.17 (2.87)	< 0.001	16.90 (2.11)	15.32 (2.77)	< 0.001

Values are mean (SD). *P* values are based on independent Student’s *t* test; (n/y: no vs. yes category)

^a^Experiencing at least one of the physical illnesses including hypertension, cardiovascular, osteoporosis, diabetic, Arthritis & digestive diseases

^b^Experiencing at least one of the mental illnesses including depression, anxiety, Parkinson, sleep disorders & Alzheimer and cognitive impairments

### Criterion validity and convergent validity

Criterion validity revealed significant positive correlations between the OPQOL-brief sub-scales and different dimensions of the SF-36 (*P* < 0.001). The Pearson correlations for the SF-36 with the total score of OPQOL-brief was r: 0.694 (*P* < 0.001), with the socioeconomic well-being was r: 0.534 (*P* < 0.001), with emotional well-being was r: 0.627 (*P* < 0.001), and with physical well-being was r: 0.637 (*P* < 0.001). Convergent validity showed significant negative correlations between the OPQOL-brief sub-scales and ISI and PSQI measures (*P* < 0.01, Table 5).

**Table 5.** Correlations of Persian OPQOL-brief 's subscales with sleep quality and SF-36 questionnaires to assess the criterion and convergent validity

	Socioeconomic well-being	Emotional well-being	Physical well-being	Total Score
ISI score	−0.379	−0.299	−0.381	−0.462
PSQI score	−0.197	−0.328	−0.218	−0.346
SF-36 score	0.534	0.627	0.637	0.694
Physical functioning	0.353	0.441	0.510	0.491
Role functioning/physical	0.407	0.445	0.482	0.518
Role functioning/emotional	0.427	0.524	0.468	0.536
Vitality	0.495	0.609	0.515	0.621
Emotional well-being	0.426	0.523	0.337	0.493
Social functioning	0.495	0.487	0.609	0.618
Bodily pain	0.395	0.468	0.575	0.555
General health	0.464	0.591	0.592	0.629
Physical component	0.482	0.559	0.637	0.650
Mental component	0.533	0.633	0.572	0.668

All correlations are significant at *P* < 0.01 level

### Reliability analyses

The reliability and descriptive statistics for the OPQOL-brief questionnaire are shown in Table 6. The ICC coefficient for the total score of the OPQOL-brief suggests good test–retest reliability (ICC = 0.842, 95% CI: 0.729 to 0.910; *P* < 0.001). The ICC coefficients for the extracted subscales including “socioeconomic”, “emotional”, and “physical” well-being were estimated to be 0.83, 0.79 and 0.73, respectively. In addition, Cronbach’s alpha coefficient to indicate item internal consistency for each sub-scale is presented in Table 6 and all scales showed satisfactory results, so that all sub-scales met or exceeded the 0.70 level recommended. The Cronbach’s alpha coefficient for the total score of the OPQOL-brief was 0.829. In addition, the percentage of respondents scoring at the lowest level (i.e., floor effect) and the highest level (i.e., ceiling effect) was minimal for all sub-scales. Distribution of individual OPQOL-brief items in studied population is also presented in the Table 7. Approximately all the item-total correlations of the Persian version of OPQOL-brief scale exceed the acceptable threshold of 0.30. They ranged from 0.241 (Item 2: I look forward to things) to 0.623 (Item 8: I feel lucky compared to most people). According to Table 7, Cronbach’s alpha for Persian OPQOL-brief was not increased if any of the items was deleted. This means that all items of questionnaire should be retained.

**Table 6.** Descriptive statistics and reliability statistics for the Persian OPQOL-brief

	Mean (SD)	Cronbach’s α	ICC (%95CI)	Floor (%)	Ceiling (%)
Socioeconomic well-being	18.11 (3.18)	0.734	0.833 (0.716–0.905)	1 (2.2)	4 (8.9)
Emotional well-being	19.44 (2.41)	0.739	0.789 (0.646–0.878)	1 (2.2)	1 (2.2)
Physical well-being	15.47 (1.99)	0.745	0.733 (0.562–0.844)	2 (4.4)	1 (2.2)
Total score	48.96 (6.32)	0.829	0.842 (0.729–0.910)	1 (2.2)	1 (2.2)

*ICC* intra class coefficient; All ICC are significant at *P* < 0.001 level

**Table 7.** Distribution of individual OPQOL-brief items in studied population and reliability statistics

	Strongly disagree	Disagree	Neither agree nor disagree	Agree	Strongly agree	Mean (SD)	Item-total correlation	Cronbach’s alpha if item deleted
I enjoy my life overall	15 (2.9)	73 (13.9)	55 (10.5)	316 (60.3)	65 (12.4)	3.65 (0.97)	0.489	0.797
I look forward to things	12 (2.3)	42 (8.0)	76 (14.6)	327 (62.6)	65 (12.5)	3.74 (0.86)	0.241	0.817
I am healthy enough to get out and about	5 (1.0)	44 (8.5)	36 (6.9)	292 (56.3)	142 (27.4)	4.01 (0.88)	0.431	0.802
My family, friends or neighbors would help me if needed	6 (1.2)	62 (11.9)	29 (5.6)	200 (38.4)	224 (43.0)	4.10 (1.03)	0.317	0.814
I am healthy enough to have my independence	6 (1.1)	45 (8.6)	25 (4.8)	291 (55.7)	155 (29.7)	4.05 (0.88)	0.537	0.793
I can please myself what I do	6 (1.1)	10 (1.9)	49 (9.4)	324 (62.0)	134 (25.6)	4.09 (0.73)	0.461	0.800
I feel safe where I live	1 (0.2)	18 (3.4)	13 (2.5)	179 (34.3)	311 (59.6)	4.50 (0.73)	0.514	0.796
I get pleasure from my home	7 (1.3)	21 (4.0)	11 (2.1)	175 (33.5)	308 (59.0)	4.45 (0.83)	0.531	0.793
I take life as it comes and make the best of things	2 (0.4)	9 (1.7)	20 (3.8)	255 (48.9)	235 (45.1)	4.36 (0.67)	0.273	0.812
I feel lucky compared to most people	7 (1.3)	48 (9.2)	95 (18.1)	280 (53.4)	94 (17.9)	3.78 (0.89)	0.623	0.785
I have enough money to pay for household bills	3 (0.6)	28 (5.4)	21 (4.0)	335 (64.5)	132 (25.4)	4.09 (0.75)	0.406	0.803
I have social or leisure activities/hobbies that I enjoy doing	14 (2.7)	43 (8.2)	39 (7.5)	276 (52.9)	150 (28.7)	3.98 (0.95)	0.514	0.794
I try to stay involved with things	5 (1.0)	35 (6.7)	41 (7.9)	313 (60.2)	126 (24.2)	4.01 (0.81)	0.534	0.793

## Discussion

In current study, the psychometric properties (test–retest reliability, internal consistency, discriminant, construct, criterion and convergent validity) of the Persian version of OPQOL-brief were evaluated. To the best of our knowledge, the OPQOL-brief is one of the few Persian versions of fully validated questionnaires to measure quality of life in Iranian older people. Internal consistency and test–retest reliability of the Persian version of OPQOL-brief were acceptable. Patient participants in our study reported lower levels of QoL than healthy older people, indicating good discriminant validity. Applying factor analysis for evaluating the construct validity led to three factors (“socioeconomic well-being”, “emotional well-being”, and “physical well-being”) in terms of QoL level. The Persian version of OPQOL-brief also showed satisfactory criterion and convergent validity.

We found that the Persian version of OPQOL-brief was satisfactory reliable. Reliability in the current study was evaluated by Cronbach’s alpha coefficients and ICC. Cronbach’s alpha coefficient for the total score of the OPQOL-brief was 0.829. This is a similar result with Cronbach’s alpha of original OPQOL-brief which was 0.856 [7], and also with the Turkish version of OPQOL-brief which was 0.867 [3]. The ICC value for the total score of the Persian version of OPQOL-brief was 0.842 and it demonstrated good stability. However, the Turkish version of OPQOL-brief showed excellent reliability with ICC value of 0.98 [3]. ICC value for the original OPQOL-brief is not reported. Approximately all the item-total correlations of the Persian version of OPQOL-brief scale exceed the acceptable threshold of 0.30. They ranged from 0.241 to 0.623. In original version of OPQOL-brief, item-total correlations ranged from 0.36 to 0.67 [7], and in Turkish version ranged from 0.349 to 0.726 [3]. The different correlations could be a result of sociocultural dissimilarity of populations and different sample size.

According to our results, the Persian version of OPQOL-brief well discriminated healthy older individuals and patients; in which QoL level was significantly lower among patients. This result supported the discriminant validity of the Persian OPQOL-brief. In line with our results, Turkish older people with multi-morbidity scored lower in the OPQOL-brief [3].

In the current study, the evaluation of construct validity of the OPQOL-brief led to extraction of three factors and three classes by EFA and LCA, respectively. The factor structure of the Persian OPQOL-brief was not consistent with other versions of this questionnaire. Principal components analysis on the original and Turkish versions of the questionnaire showed that the OPQOL-brief is a uni-dimensional scale [3, 7]. Similar to the aforesaid versions, factor loadings for all 13 items of the Persian OPQOL-brief exceeded 0.40. The construct validity of the OPQOL-brief was not evaluated in other versions using LCA. The divergent findings could be attributed to differences in socio-economic status and culture of studied populations.

In our study, correlation analysis was performed between the Persian OPQOL-brief and SF-36 for evaluating criterion validity. As expected, there were moderate to high correlations between two questionnaires and their sub-scales. In addition, ISI and PSQI scales which measure sleep quality of an individual were significantly and inversely correlated with the Persian OPQOL-brief scores. These findings indicate appropriate criterion and convergent validities of the Persian OPQOL-brief. In line with our results, positive correlation was observed between CASP-19 and Turkish OPQOL-brief scales [3].

There were some limitations in our study. We selected the sample only from Isfahan (located at central of Iran); therefore the representativeness of this sample for all Iranian older people or other Persian language countries should be interpreted with caution. We used the self-report questionnaire to assess the participants' health status.

## Conclusions

The findings suggest that the Persian version of OPQOL-brief questionnaire is a reliable and valid measure for evaluating the quality of life in Persian-speaking older adults. The OPQOL-brief is based on perspectives and own thoughts of older people, easy to understand and takes nearly 15 min to be completed.

## Supplementary information

**Additional file 1: Table S1**. Class-specific levels of quality of life items, and the size of classes based on Latent Class Analysis (LCA).

## Data Availability

Not applicable.

## Abbreviations

OPQOL-brief: The brief Older People’s Quality of Life questionnaire
QoL: Quality of life
ICC: Intra Class Correlation
EFA: Exploratory factor analysis
CFA: Confirmatory factor analysis
LCA: Latent class analysis
KMO: Kaiser–Meyer–Olkin
AIC: Akaike's information criterion
BIC: Bayesian information criteria
PF: Physical functioning
RP: Role limitations due to physical health
BP: Body pain
GH: General health
MH: Mental health
RE: Role limitations due to emotional problems
VT: Vitality (energy/fatigue)
SF: Social functioning
PSQI: Pitsburgh Sleep Quality Index
ISI: Insomnia Severity Index
