# Supplementary material for: Persian version of the brief Older People’s Quality of Life questionnaire (OPQOL-brief): the evaluation of the psychometric properties
Source: Health Qual Life Outcomes. 2020 Oct 7;18:327. doi: 10.1186/s12955-020-01586-8 (PMC7542137; doi:10.1186/s12955-020-01586-8)
Supplement: Supplementary file 1 — Additional file 1: Table S1. Class-specific levels of quality of life items, and the size of classes based on Latent Class Analysis (LCA). [file 12955_2020_1586_MOESM1_ESM.docx]

**Supplementary files**

| **Table S1. Class-specific levels of quality of life items, and the size of classes** | | | | |
| --- | --- | --- | --- | --- |
|  | | **Class1:**  **Low quality of life** | **Class2:**  **Middle quality of life** | **Class3:**  **High quality of life** |
| **Class Size (%)** | | **16%** | **67%** | **17%** |
| **I enjoy my life overall** | |  |  |  |
|  | **Strongly disagree** | **0.12** | **0.01** | **0.00** |
|  | **Disagree** | **0.37** | **0.11** | **0.00** |
|  | **Neither agree nor disagree** | **0.15** | **0.12** | **0.02** |
|  | **Agree** | **0.33** | **0.67** | **0.57** |
|  | **Strongly agree** | **0.02** | **0.08** | **0.41** |
| **I look forward to things** | |  |  |  |
|  | **Strongly disagree** | **0.04** | **0.02** | **0.00** |
|  | **Disagree** | **0.13** | **0.09** | **0.01** |
|  | **Neither agree nor disagree** | **0.19** | **0.16** | **0.04** |
|  | **Agree** | **0.58** | **0.64** | **0.61** |
|  | **Strongly agree** | **0.06** | **0.08** | **0.34** |
| **I am healthy enough to get out and about** | |  |  |  |
|  | **Strongly disagree** | **0.04** | **0.00** | **0.00** |
|  | **Disagree** | **0.27** | **0.06** | **0.00** |
|  | **Neither agree nor disagree** | **0.13** | **0.07** | **0.00** |
|  | **Agree** | **0.49** | **0.65** | **0.26** |
|  | **Strongly agree** | **0.07** | **0.22** | **0.73** |
| **My family, friends or neighbors would help me if needed** | |  |  |  |
|  | **Strongly disagree** | **0.05** | **0.00** | **0.00** |
|  | **Disagree** | **0.36** | **0.08** | **0.02** |
|  | **Neither agree nor disagree** | **0.10** | **0.05** | **0.03** |
|  | **Agree** | **0.33** | **0.41** | **0.33** |
|  | **Strongly agree** | **0.15** | **0.46** | **0.61** |
| **I am healthy enough to have my independence** | |  |  |  |
|  | **Strongly disagree** | **0.06** | **0.00** | **0.00** |
|  | **Disagree** | **0.30** | **0.05** | **0.00** |
|  | **Neither agree nor disagree** | **0.10** | **0.05** | **0.00** |
|  | **Agree** | **0.49** | **0.69** | **0.10** |
|  | **Strongly agree** | **0.05** | **0.21** | **0.90** |
| **I can please myself what I do** | |  |  |  |
|  | **Strongly disagree** | **0.06** | **0.00** | **0.00** |
|  | **Disagree** | **0.07** | **0.01** | **0.00** |
|  | **Neither agree nor disagree** | **0.20** | **0.09** | **0.00** |
|  | **Agree** | **0.61** | **0.72** | **0.19** |
|  | **Strongly agree** | **0.05** | **0.18** | **0.81** |
| **I feel safe where I live** | |  |  |  |
|  | **Strongly disagree** | **0.01** | **0.00** | **0.00** |
|  | **Disagree** | **0.17** | **0.01** | **0.00** |
|  | **Neither agree nor disagree** | **0.08** | **0.02** | **0.00** |
|  | **Agree** | **0.52** | **0.38** | **0.03** |
|  | **Strongly agree** | **0.21** | **0.60** | **0.97** |
| **I get pleasure from my home** | |  |  |  |
|  | **Strongly disagree** | **0.07** | **0.00** | **0.00** |
|  | **Disagree** | **0.20** | **0.01** | **0.00** |
|  | **Neither agree nor disagree** | **0.07** | **0.02** | **0.00** |
|  | **Agree** | **0.47** | **0.39** | **0.02** |
|  | **Strongly agree** | **0.20** | **0.58** | **0.98** |
| **I take life as it comes and make the best of things** | |  |  |  |
|  | **Strongly disagree** | **0.01** | **0.00** | **0.00** |
|  | **Disagree** | **0.03** | **0.02** | **0.00** |
|  | **Neither agree nor disagree** | **0.06** | **0.04** | **0.00** |
|  | **Agree** | **0.58** | **0.55** | **0.22** |
|  | **Strongly agree** | **0.33** | **0.39** | **0.78** |
| **I feel lucky compared to most people** | |  |  |  |
|  | **Strongly disagree** | **0.07** | **0.00** | **0.00** |
|  | **Disagree** | **0.37** | **0.04** | **0.00** |
|  | **Neither agree nor disagree** | **0.33** | **0.19** | **0.01** |
|  | **Agree** | **0.23** | **0.65** | **0.37** |
|  | **Strongly agree** | **0.01** | **0.11** | **0.62** |
| **I have enough money to pay for household bills** | |  |  |  |
|  | **Strongly disagree** | **0.02** | **0.00** | **0.00** |
|  | **Disagree** | **0.16** | **0.04** | **0.00** |
|  | **Neither agree nor disagree** | **0.08** | **0.04** | **0.00** |
|  | **Agree** | **0.65** | **0.72** | **0.33** |
|  | **Strongly agree** | **0.09** | **0.20** | **0.67** |
| **I have social or leisure activities/hobbies that I enjoy doing** | |  |  |  |
|  | **Strongly disagree** | **0.14** | **0.00** | **0.00** |
|  | **Disagree** | **0.33** | **0.03** | **0.00** |
|  | **Neither agree nor disagree** | **0.16** | **0.07** | **0.01** |
|  | **Agree** | **0.34** | **0.62** | **0.38** |
|  | **Strongly agree** | **0.04** | **0.27** | **0.61** |
| **I try to stay involved with things** | |  |  |  |
|  | **Strongly disagree** | **0.06** | **0.00** | **0.00** |
|  | **Disagree** | **0.30** | **0.02** | **0.00** |
|  | **Neither agree nor disagree** | **0.20** | **0.07** | **0.00** |
|  | **Agree** | **0.43** | **0.73** | **0.29** |
|  | **Strongly agree** | **0.02** | **0.18** | **0.71** |
